# Impaired smooth muscle cell contractility as a novel concept of abdominal aortic aneurysm pathophysiology

**DOI:** 10.1038/s41598-019-43322-3

**Published:** 2019-05-02

**Authors:** Natalija Bogunovic, Jorn P. Meekel, Dimitra Micha, Jan D. Blankensteijn, Peter L. Hordijk, Kak K. Yeung

**Affiliations:** 1 Departments of Vascular Surgery, Amsterdam University Medical Centers, location VU University Medical center, Amsterdam, The Netherlands; 2 Departments of Physiology, Amsterdam University Medical Centers, location VU University Medical center, Amsterdam, The Netherlands; 3 Departments of Clinical Genetics, Amsterdam University Medical Centers, location VU University Medical center, Amsterdam, The Netherlands

**Keywords:** Preclinical research, Translational research

## Abstract

Ruptured abdominal aortic aneurysms (AAA) are associated with overall mortality rates up to 90%. Despite extensive research, mechanisms leading to AAA formation and advancement are still poorly understood. Smooth muscle cells (SMC) are predominant in the aortic medial layer and maintain the wall structure. Apoptosis of SMC is a well-known phenomenon in the pathophysiology of AAA. However, remaining SMC function is less extensively studied. The aim of this study is to assess the *in vitro* contractility of human AAA and non-pathologic aortic SMC. Biopsies were perioperatively harvested from AAA patients (n = 21) and controls (n = 6) and clinical data were collected. Contractility was measured using Electric Cell-substrate Impedance Sensing (ECIS) upon ionomycin stimulation. Additionally, SMC of 23% (5 out of 21) of AAA patients showed impaired maximum contraction compared to controls. Also, SMC from patients who underwent open repair after earlier endovascular repair and SMC from current smokers showed decreased maximum contraction vs. controls (p = 0.050 and p = 0.030, respectively). Our application of ECIS can be used to study contractility in other vascular diseases. Finally, our study provides with first proof that impaired SMC contractility might play a role in AAA pathophysiology.

## Introduction

Abdominal aortic aneurysms (AAA) are considered among the most severe surgical emergencies due to the overall mortality rate of 90% in case of rupture^1^. The chance of rupture increases with a larger AAA size and/or high growth rate^2,3^. Given that AAA cases mostly are not consequences of a specific traumatic event, infection or a genetic connective-tissue disorder, the majority of AAA cases are considered non-specific or sporadic^3^. Even though the pathophysiology of aortic aneurysms remains unclear, the disease is associated with atherosclerosis, tobacco smoking and male gender^3^. The lack of a known molecular mechanism increases the difficulties of finding targets for pharmacological therapies, which leaves surgical intervention as the only available treatment option^4^. Smooth muscle cells (SMC) are the predominant cell type in the aortic media, which represents the thickest layer of the aorta. SMCs are oriented radial and, using their contractile properties, regulate blood flow and pulse pressure in the aorta^5^. Furthermore, SMC contractile units contribute to force distribution in the aorta by regulating the mechanical properties through their link with the extracellular matrix^6^. Mutations in genes encoding for contractile proteins, such as smooth muscle myosin heavy chain (*MYH11*) and smooth muscle actin (*ACTA2*) have been associated with cases of hereditary thoracic aortic aneurysms and dissections^5,6^. This underscores a potential role of SMC contractile elements in aortic aneurysmal pathology^6–8^. However, little is known regarding the SMC function, especially the role of SMC contraction in sporadic AAA.

Measuring SMC contraction is challenging. *In vitro* SMC contractility and associated signaling have so far mostly been measured indirectly by traction force microscopy^9,10^, quantification of Fura-2 fluorescence intracellular calcium fluxes^11^ and collagen wrinkling assays^12^. Although indispensable for the gain of knowledge of SMC function in culture, most available assays are low throughput and therefore not optimal for screening of patient SMC biobanks. To overcome this problem, we chose to use a new method: the electric cell-substrate impedance sensing (ECIS). ECIS is a real-time, medium throughput assay, widely used to quantify adherent cell behavior and contraction^13–16^. ECIS has been used previously to study SMC growth and behavior in wound-healing and migration assays^17–19^. We thus opted to use ECIS as a novel, quantitative strategy to analyze the contractile responses of vascular SMC.

To examine the role of SMC contractility in AAA pathophysiology, we measured the contractile properties of SMC isolated from aortic biopsies of controls and sporadic AAA patients. Using the ECIS, we compared the contractile properties of SMC derived from biopsies of both non-ruptured and ruptured AAA and correlated our findings with clinical characteristics and SMC-marker expression profile of the patients. The aim of this study is to evaluate SMC contractility in patients with sporadic AAA.

## Results

### Smooth muscle cell contraction

**Figure 1:**
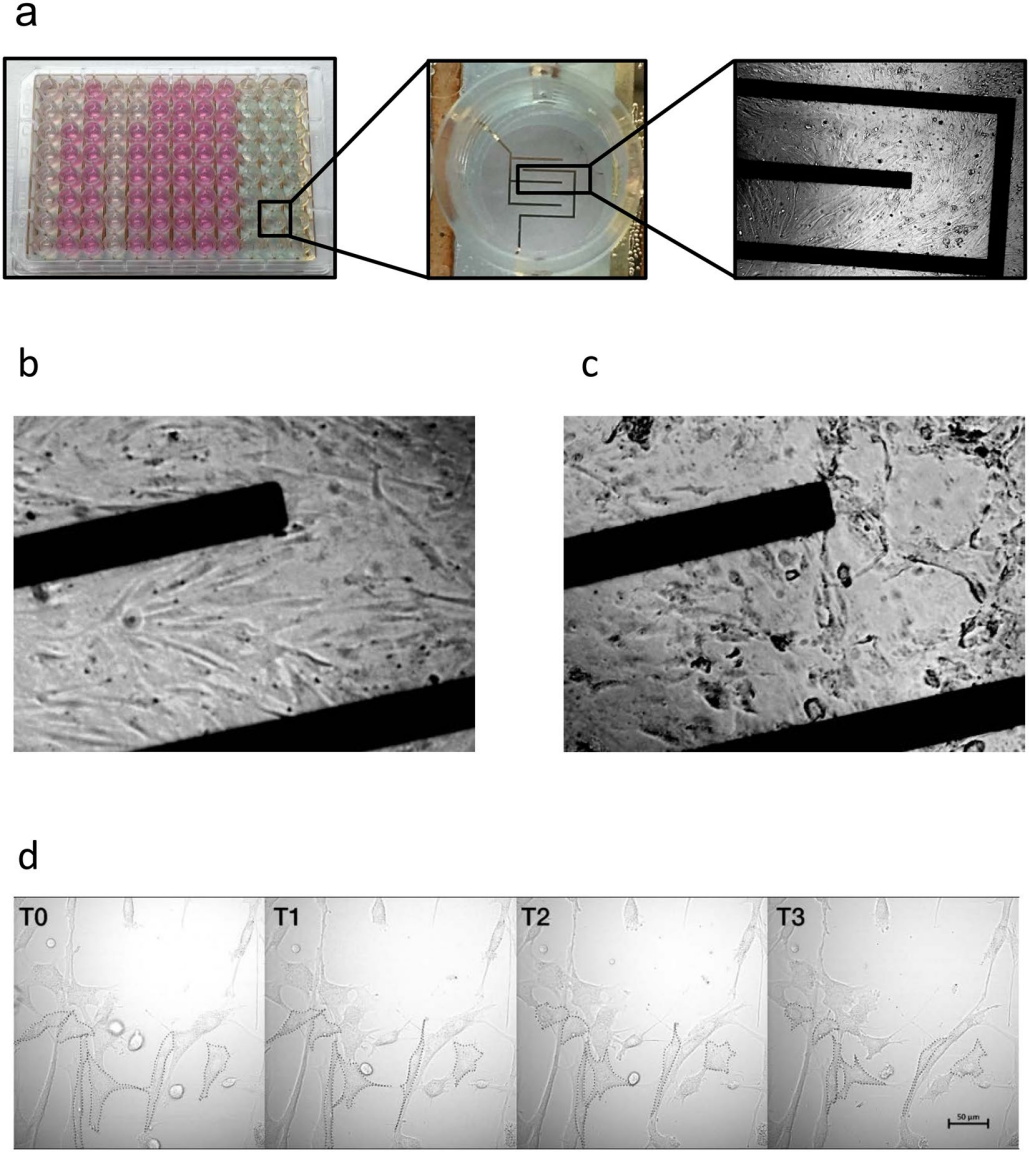
Graphic representation of aortic SMC contraction. (**a**) Left; ECIS 96e10e cultureware plate. Middle; magnified picture of a single well within the plate with a detailed view of the ten electrodes on the bottom of the well. Right; light microscope image of SMC seeded on the plate. (**b**) Representative images of a monolayer of control SMC prior to stimulation of contraction. (**c**) Representative images of a monolayer of control SMC post stimulation. (**d**) Representative images of control SMC contraction captured by time-lapse microscopy. T0 image depicts cells prior to stimulation, and T1-3 depict time points post stimulation. The outline of five representative cells is marked with dotted lines to represent the change in cell shape during contraction. Scale bar: 50 µm.

**Figure 2:**
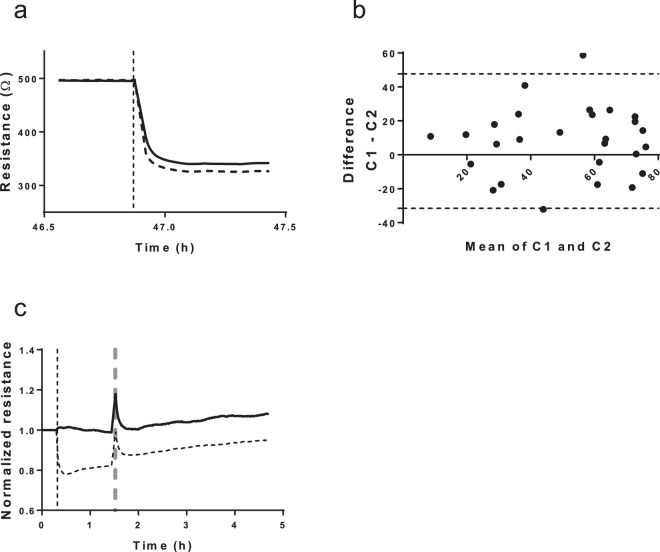
Measuring SMC contraction using ECIS. (**a**) Representative intraexperimental reproducibility of ECIS contraction measurements. Resistance curves generated by SMC of control 1 seeded in two wells. Vertical dotted line marks the stimulation. (**b**) Difference plot demonstrating interexperimental measurement reproducibility between two separate contraction measurements. Dotted lines represent the 95% confidence interval and thick medial line represents the mean of two interexperimental measurements. Each data point represents the deviation from the mean of two independent measurements. (**c**) Cell recovery post stimulation of contraction. Black thick line represents the unstimulated resistance value of a control smooth muscle cell line. Dotted line represents the stimulated resistance value of the same cell line. Resistance values were normalized to the values pre stimulation to monitor the behavior of cells post stimulation. Vertical dotted line marks the time of stimulation. After approximately 1 h post stimulation, the medium was refreshed to remove the stimulus (vertical dotted grey line) and the recovery of the cells was tracked for a few hours longer.

Contractility of aortic smooth muscle cells could be quantified using the ECIS. Adherent SMC, seeded on gold plated electrodes (Fig. 1a), were stimulated with ionomycin to induce a contractile response within a few seconds. As depicted in Fig. 1c, the stimulated cells contracted and lost cell-cell contact post stimulation, compared to the same monolayer in Fig. 1b. The consequent reduction in surface coverage is measured by ECIS as a drop in impedance. This way, SMC contraction can be quantified using ECIS, as deduced from the almost immediate and significant decrease of impedance post stimulation. The same process can be observed in Fig. 1d, where a monolayer of SMC shows contraction in a time-lapse recording. The marked cell outlines of five representative cells indicates the change in cell shape during contraction. The full time-lapse video is available as Supplementary Video 1. Intraexperimental reproducibility is shown on Fig. 2a, where the two curves represent two stimulated wells of control 1. Vertical dotted line marks the time point on the x axes which indicates stimulation with ionomycin and consequent reduction of resistance which corresponds to contraction. A representative interexperimental difference plot (Bland-Altman) shows the reproducibility between independent contraction measurements in control and patient SMC. As depicted in Fig. 2b, there are two outliers in the combined group of 27 controls and patients. The vast majority of contractility measurements post stimulation are within the 95% confidence interval. Cell recovery post stimulation of contraction is depicted in Fig. 2c. Black thick line represents the unstimulated resistance value of a control smooth muscle cell line. Dotted line represents the stimulated resistance value of the same cell line. Resistance values were normalized to the values pre stimulation to monitor the behavior of cells post stimulation. Vertical dotted line marks the time point on the x-axes, which indicates stimulation with ionomycin and consequent reduction of resistance which corresponds to contraction. After approximately 1 h post stimulation, the medium was refreshed to remove the stimulus (vertical dotted grey line) and the recovery of the cells was tracked for the next few hours.

### Contractile response of control and AAA patient’ SMC

**Figure 3:**
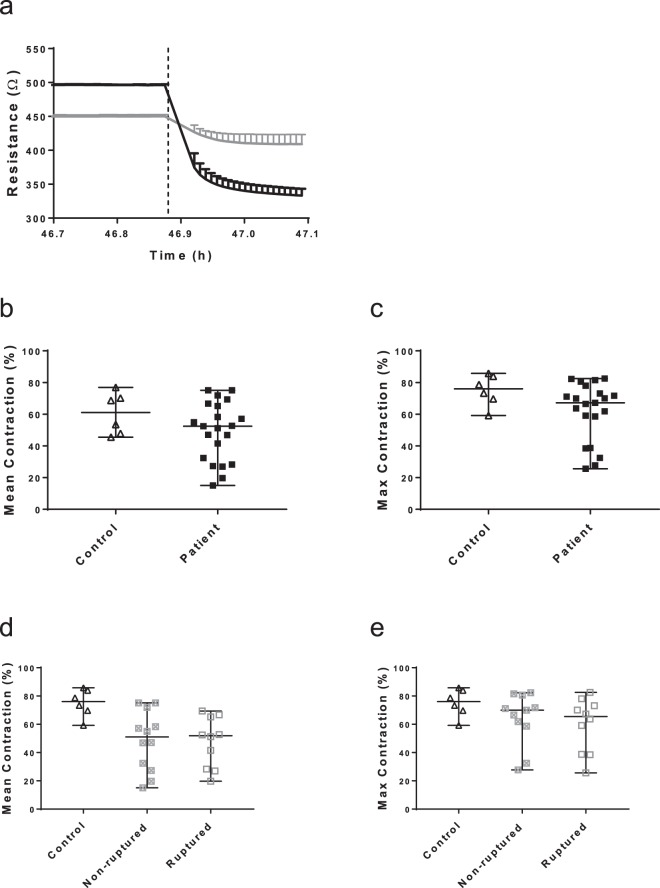
Contraction of control and AAA patient’ SMC upon ionomycin stimulation. (**a**) Representative resistance curves generated by control 1 (black) and patient 1 (grey) SMC before and after stimulation of contraction. Resistance values were measured in duplicate and represented as mean with SD. Vertical dotted line marks the stimulation with ionomycin. (**b**) Mean contractile response of control (▲; n = 6) and AAA patient (■; n = 21) SMC upon ionomycin stimulation derived from multiple experiments. (**c**) Maximum contractile response of control (▲; n = 6) and AAA patients’ (■; n = 21) SMC upon ionomycin stimulation. (**d**) Mean contractile response of control (▲; n = 6), non-ruptured AAA patients’ (⊠; n = 13) and ruptured AAA patients’ (□; n = 7) SMC from multiple experiments. (**e**) Maximum contractile response of control (▲; n = 6), non-ruptured AAA patients’ (⊠; n = 13) and ruptured AAA patients’ (□; n = 7) SMC. Contraction is expressed in percentages of decrease compared to baseline value. Boxplots are shown as median with range.

Contractility of SMC isolated from the aortic biopsies of controls and sporadic AAA patients was quantified using the ECIS. Resistance curves of control 1 and patient 1 are depicted in Fig. 3a, representing exemplary normal contraction in the control and impaired contraction in the patient group. The dotted line marks the ionomycin stimulation. Resistance values decrease post stimulation and a steeper curve can be noted in the control compared to the patient cell line. The median of the average contractile response of the control group (n = 6) was 61% (46–77%) of the initial value pre-stimulation vs. 52% (15–75%) for the patient group (Fig. 3b). The median of the maximum contractile response of the control group (n = 6) was 76% (59–86%) of the pre-stimulation value vs. 67% (26–83%) for the patient group (Fig. 3c).

The contractile response of the patients’ cells exhibited larger variability. We could not detect a significant difference between all AAA patients and the control group, neither in mean (p = 0.235) nor in maximum contraction (p = 0.085). No significant difference was found between the mean contractile response of the cells from patients with non-ruptured aneurysms (median response 54%, range: 15–75%, p = 0.525) vs. cells from those with ruptured aneurysms (median response 51%, range: 20–69%; p = 0.417; Fig. 3d). In addition, no significant differences in maximum contraction were found between controls vs. SMC derived from non-ruptured aneurysms (median response 69%, range: 28–82%, p = 0.216) vs. cells from those with ruptured aneurysms (median response 65%, range: 26–83%; p = 0.73; Fig. 3e). The median responses of both non-ruptured and ruptured AAA groups were almost identical and heterogeneous like the response spread of the cells from all AAA patients. Mean and maximum contraction of all 27 cell lines was significantly correlated (R = 0.853, p < 0.001).

**Figure 4:**
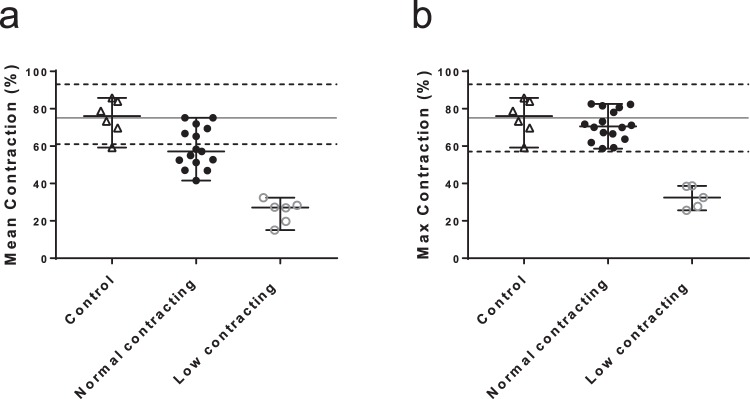
Contractile response of control and AAA patient’ SMC. (**a**) Mean contractile response of Control (▲; n = 6), Normal contracting (●; n = 15) and Low contracting (○; n = 6) AAA patients’ SMC derived from multiple experiments. (**b**) Maximum contractile response of Control (▲; n = 6), Normal contracting (●; n = 16) and Low contracting (○; n = 5) AAA patients’ SMC. Black horizontal line marks the mean of contraction of the control group. Dotted horizontal lines mark two SD higher and lower than the mean of the control group. Low contracting group is defined as contraction lower than two SD bellow the mean of the control group. Contraction is expressed in percentages of decrease compared to baseline value. Boxplot is shown as median with range.

Using the control group as a reference for normal SMC contractility *in vitro*, the patient group was divided into low and high contracting groups. Patient cell lines exhibiting contractility lower than two standard deviations below the mean of the controls, i.e. a mean contractile response lower than 34% and a maximum contractile response lower than 57%, were categorized as low contracting. Upon screening both ruptured and non-ruptured patient SMC, 28% of the AAA-patients (ruptured and non-ruptured) SMC contractile response showed repeatedly lower contractility (Low contracting; n = 6/21, Fig. 4a). The low contracting AAA patients’ cells thus showed a median contractile response of 35% (26–59%). The same trend could be observed in the measurements of maximum contraction; where 23% of all AAA patient SMC (5 out of 21) exhibited lower contractility (median 32%, range 26–39%, Fig. 4b). The other SMC from the majority of AAA patients contracted in the same range as the controls, with a mean response of 57% (42–75%; n = 15) and maximum response of 70% (59–83%). The patients with cells with low contractility belonged to both the ruptured and non-ruptured AAA groups.

### Contraction of cells from AAA patients and SMC phenotype

**Figure 5:**
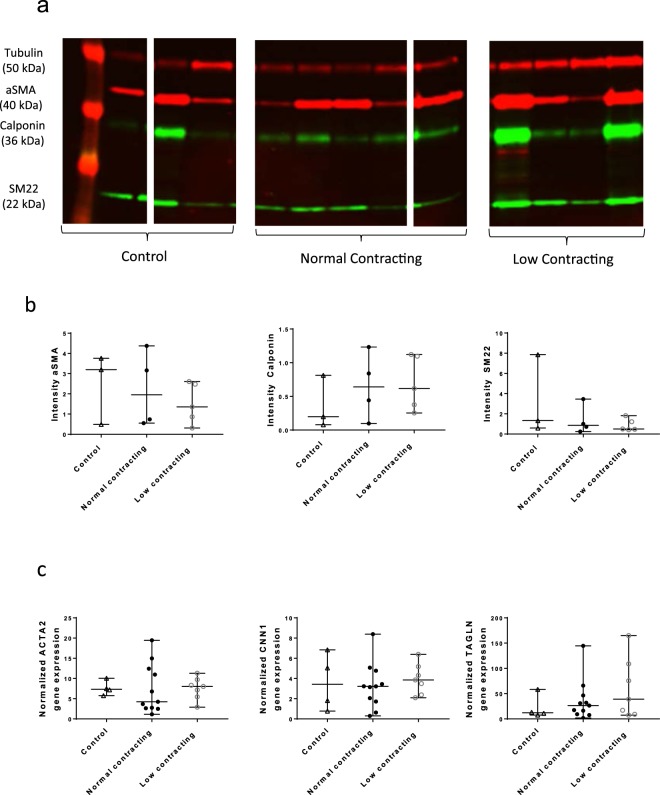
Expression of SMC phenotypic markers. (**a**) Western blot analysis of aSMA, Calponin and SM22 in Control (n = 3), Normal Contracting (n = 5) and Low Contracting (n = 4) AAA patients’ SMC. Lanes were cropped and grouped from the original image (Supplementary Figure 3), Ladder: lane 0; Control: lanes 1,4 and 5; Normal contracting: lanes 6–9 and 14; Low contracting: lanes 10–13. (**b**) Intensity of aSMA, Calponin and SM22 in Control (n = 3), Normal Contracting (n = 5) and Low Contracting (n = 4) AAA patients’ SMC. Intensity red channel (700CW) 5, Intensity green channel (800CW) 3. (**c**) Gene expression of *ACTA2, CNN1 and TAGLN* in mRNA isolated from control (▲; n = 4), normal contracting (●; n = 13) and low contracting SMC (○; n = 5). Boxplots are shown as median with range.

To compare the phenotypic state of Control vs. Normal and Low contracting SMC, changes in SMC specific marker gene expression were studied using qPCR. Firstly, we compared the protein expression of SMC marker proteins aSMA, Calponin and SM22 using western blot (Fig. 5a). As shown in the quantification of the blot in Fig. 5b, the expression of SMC markers is very heterogeneous in the control as well as in both patient contraction groups. Similar trend of variability can be observed on mRNA level, with the expression of the corresponding SMC marker genes, *ACTA2, CNN1* and *TAGLN* (Fig. 5c).

Additionally, no significant differences in gene expression of *SMTN* and *VIM* were observed between cells from controls and Normal and Low contracting patient SMC (Supplementary Figure 2). Furthermore, no significant difference in expression levels of *MKi67* between cells from controls and Normal and Low contracting patient SMC (Supplementary Figure 2) was found, indicating no difference in proliferative capacity. In addition, no differences were found between marker expressions in controls and all patients or controls and sub-groups of non-ruptured and ruptured AAA patient SMC.

To examine overall cell morphology and cytoskeletal properties, as well as expression of the contractile phenotype, immunostaining for smoothelin and F-actin (Supplementary Figure 1) was performed in fixed cells of two controls and three patients. We observed spindle shaped, adherent SMC with long actin fibers and consistent expression of smoothelin, confirming that both control and patient cell lines express this marker of mature SMC. Cell morphology and marker expression do not seem to indicate a phenotypic defect linked to SMC contraction.

### Contraction of cells from AAA patients vs. clinical characteristics

**Figure 6:**
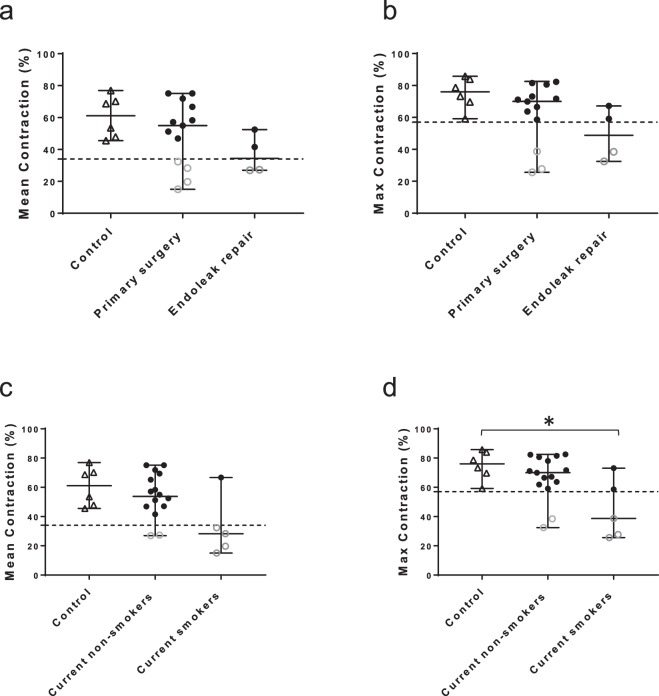
Correlation between impaired contraction of AAA patient SMC and clinical characteristics. (**a**) Mean contractile response of Control (▲; n = 6), patients who underwent primary aneurysm repair (Primary surgery; n = 17) and patients who underwent secondary surgery for endoleak repair (Endoleak repair; n = 4) derived from multiple experiments. (**b**) Maximum contractile response of Control (▲; n = 6), patients who underwent primary aneurysm repair (Primary surgery; n = 17) and patients who underwent secondary surgery for endoleak repair (Endoleak repair; n = 4). SMC obtained during endoleak repair exhibit a trend of lower contractility compared to control group (p = 0.050). (**c**) Mean contractile response of Control (▲; n = 6), Current non-smokers; n = 16 and Current smokers; n = 5 derived from multiple experiments. (**d**) Maximum contractile response of Control (▲; n = 6), Current non-smokers; n = 16 and Current smokers; n = 5. Patients with normal contraction are marked with ● and patients with low contraction (more than 2 SD bellow the mean of the control group) are marked with ○. SMC of patients who currently smoke exhibit impaired contractility compared to the control group (p = 0.030). Boxplots are shown as median with range and tested with Mann-Whitney U test.

To further analyze the link between contractility of SMC from AAA patients with AAA pathology, clinical characteristics (Table 1) were correlated with contractile output. No correlations were found between contraction of all control and patient SMC and gender and age. No correlation between contraction and patient gender or age, maximum aneurysm size, diabetes, hypertension, hyperlipidemia and pre-surgery statin use was found. With the critical value adjusted from 0.05 to 0.006 upon performing the Bonferroni correction, none of the parameters was significantly correlated with contraction. We compared the contractility of SMC which were obtained during open repair after earlier EVAR because of persisting endoleak (n = 4) to contractility of control SMC (n = 6) and SMC obtained during primary aneurysm open repair (n = 17). SMC isolated from reintervention surgery (post-endoleak) demonstrated a trend of lower maximum contraction vs. control, which was not statistically significant (p = 0.050) based on the small group sizes. Additionally, no significant difference was detected vs. primary aneurysm repair (Fig. 6b). A similar trend could be observed in case of mean contraction, with an overall trend of lower contraction in the post-EVAR group (Fig. 6a). Furthermore, patients who declared themselves as current smokers (n = 5/21) exhibited lower maximum contraction compared to the control group (p = 0.030) whereas the SMC of patients who currently do not smoke showed no difference vs. control group (Fig. 6d). No significant differences in mean contraction were found between the control and current smokers group (Fig. 6c).

**Table 1:** Clinical characteristics of controls and patients.

Type of biopsy donor	Patient code	Age at time of biopsy	Gender	Aneurysm size (mm)	Genetic cause	Smoking	Diabetes Mellitus	Hypertension	Hyperlipidemia	Statin use pre
Ruptured abdominal aortic aneurysm	RAAA1	72	Female	60	None	Less than 1 package/day	No	Yes	Yes	Yes
Ruptured abdominal aortic aneurysm	RAAA2	60	Male	100	None	Stopped less than 10 years ago	No	Unknown	Yes	Yes
Ruptured abdominal aortic aneurysm	RAAA3	65	Male	83	None	More than 1 package/day	Yes; adult	Yes	Yes	Yes
Ruptured abdominal aortic aneurysm	RAAA4Ɨ	79	Male	72	None	Stopped more than 10 years ago	No	No	Yes	Yes
Ruptured abdominal aortic aneurysm	RAAA5	67	Male	71	None	No	No	No	Yes	No
Ruptured abdominal aortic aneurysm	RAAA6	70	Male	55	None	No	No	Yes	No	Yes
Ruptured abdominal aortic aneurysm	RAAA7	88	Female	79	None	No	Yes; adult	Yes	No	Yes
Ruptured abdominal aortic aneurysm	RAAA8	83	Female	100	None	More than 1 package/day	No	Yes	No	No
Non-ruptured abdominal aortic aneurysm	NRAAA1Ɨ	69	Male	88	None	No	No	Yes	Yes	Yes
Non-ruptured abdominal aortic aneurysm	NRAAA2Ɨ	73	Male	86	None	No	No	Yes	No	No
Non-ruptured abdominal aortic aneurysm	NRAAA3Ɨ	72	Male	90	None	No	No	No	No	Yes
Non-ruptured abdominal aortic aneurysm	NRAAA4	75	Female	63	None	Less than 1 package/day	No	No	Yes	Yes
Non-ruptured abdominal aortic aneurysm	NRAAA5	58	Female	58	None	Less than 1 package/day	No	Yes	Yes	Yes
Non-ruptured abdominal aortic aneurysm	NRAAA6	71	Male	41	None	No	Yes; adult	Yes	Yes	Yes
Non-ruptured abdominal aortic aneurysm	NRAAA7	64	Male	63	None	Stopped more than 10 years ago	No	No	No	Yes
Non-ruptured abdominal aortic aneurysm	NRAAA8	72	Female	71	None	No	No	No	No	Yes
Non-ruptured abdominal aortic aneurysm	NRAAA9	74	Male	45	None	No	Yes; adult	Yes	Yes	Yes
Non-ruptured abdominal aortic aneurysm	NRAAA10#	76	Male	77	None	Stopped less than 10 years ago	No	Yes	No	No
Non-ruptured abdominal aortic aneurysm	NRAAA11	60	Male	56	None	stopped more than 10 years ago	Unknown	Yes	Yes	No
Non-ruptured abdominal aortic aneurysm	NRAAA12	65	Male	94	None	stopped more than10 years ago	Unknown	Yes	No	Yes
Non-ruptured abdominal aortic aneurysm	NRAAA13	68	Male	55	None	No	Unknown	Yes	No	No
Control	C1	30	Male	N/A	N/A	N/A	N/A	N/A	N/A	N/A
Control	C2	44	Female	N/A	N/A	N/A	N/A	N/A	N/A	N/A
Control	C3	45	Male	N/A	N/A	N/A	N/A	N/A	N/A	N/A
Control	C4	59	Male	N/A	N/A	N/A	N/A	N/A	N/A	N/A
Control	C5	35	Male	N/A	N/A	N/A	N/A	N/A	N/A	N/A
Control	C6	22	Male	N/A	N/A	N/A	N/A	N/A	N/A	N/A

Age and gender are shown for all controls and patients. Aneurysm related characteristics are shown for patients when available: Aneurysm size (mm), known genetic causes of aneurysm, smoking history, diabetes mellitus, hypertension, hyperlipidemia and statin use pre surgery. Patients are coded RAAA 1–8 if the tissue derived from a ruptured aneurysm, and NRAAA1–13 in case of elective aneurysm repair. Patients whose tissue was collected during open repair post-EVAR are marked with an additional + . Patient who’s tissue was collected after a suprarenal repair following an infrarenal repair is marked with #.

## Discussion

Genetic dysfunctions in SMC contractile proteins and their devastating consequences for the aortic wall have been shown in cases of familial thoracic aneurysms^5,6^. Although SMCs are implicated in the pathogenesis of AAA, due to their decreased density in the aortic media^3^ and pathological apoptosis^20^, little is known of their contractile function in the context of sporadic AAA pathogenesis. To investigate the role of SMC in AAA pathology, we employed ECIS to perform a real time screen of control and patient SMC contractility *in vitro*. Using novel methodology, we measured low SMC contraction *in vitro* in a sub-group of included patients with sporadic AAA. In addition, we show a link between impaired contractility of SMCs of AAA patients and current tobacco smoking, suggesting a possible link between the effects of present-day smoking and SMC dysfunction.

Our findings on SMC contractility *in vitro* provide the first evidence of SMC contractile dysfunction in sporadic AAA patients. In comparison to the hereditary aneurysms, both thoracic and abdominal sporadic aortic aneurysms have a broader spectrum of potential causes^3^, making AAA more difficult to study and to use in the design of future molecular therapies. Furthermore, AAA is considered to be caused by a combination of lifestyle, gender, age or other cardiovascular disorders^3^. SMC function in the aorta is mostly studied or explained through analysis of what happens during dysfunction^12^. Current literature proposes many hypotheses regarding the relevance of SMC contractility for aortic function and structure^5–8^, and the severe consequences if that function becomes impaired. However, little experimental work has been published to prove this concept and to address how and why these defects have such disruptive effects on aortic wall structure.

We investigated this concept using a novel assay to measure cell contraction, based on the use of ECIS to study SMC contraction in the context of AAA. SMC contractility was previously studied by various *in vitro* methods, focusing on elucidating the *in vitro* contractile mechanisms in physiological^9–11^ and pathophysiological settings^12^. If we compare ECIS to other contraction assays, we note that other available contraction assays such as traction force microscopy are more single cell oriented and require extensive optimization and technical possibilities. More importantly, the time of contraction cannot be predicted beforehand, making filming the contraction with the traction force microscopy difficult to perform. Additionally, since the assays focus on single or a small number of cells, it is not certain that they could be used to extrapolate larger-scale screening data of patient material. A previous *ex vivo* study of tensile forces demonstrate that healthy abdominal aortic samples exhibit higher tensile strength than *ex vivo* samples obtained during AAA open repair^21^. Their study indicates the important role of tensile strength in the aorta, and the consequences of stiffness and elasticity disbalance in individual aneurysm pathology. Moreover, SMC contractility has been investigated in thoracic aorta SMC from Marfan mice. Marfan syndrome is a connective tissue disorder caused by mutations in *FBN1*, and the most severe clinical manifestations include aortic aneurysms and dissections^22^.Thoracic aortas of Marfan mice exhibited a reduction of 64 to 81% reduced contraction upon KCl and phenylephrine compared to age matched control mice^23^. This data is in good agreement with our current findings using patient SMC. On a molecular level, it has been published that inflammasome activity leads to the degradation of SMC specific proteins in aortas from patients with sporadic thoracic aortic aneurysm^12^. Using a collagen-wrinkling assay, these authors showed that the activity of the inflammasome leads to reduced SMC contraction *in vitro*. Taken together, literature indicates a link between SMC dysfunction, in particular reduced contractility, and AAA pathology.

Previous studies investigated the link between aneurysm wall stress and risk of rupture, demonstrating a link between increased wall stress and risk of rupture in risk prediction models^24,25^. Strikingly, in our assay the clinically distinct groups do not differ in heterogeneity nor in median contractile response. This indicates that the reduced contractility is neither a cause nor a consequence of AAA rupture.

One limitation of this study is the relatively small patient sample and the discrepancy in size between the patient and control groups. However, due to the homogeneity of the control contractile responses in our assay, we present the findings within the current sample size. The small group sizes might account for the lack of correlation between contraction and important clinical parameters, such as age and aneurysm size. An additional limitation is the use of cells obtained through an explant protocol. This means that the cells used in the experiments proliferated from the aortic biopsy which might influence their properties or decrease the effects present in the same cells *in vivo*. Moreover, the contractile division into high and low contracting patient cells might be affected by the anatomical region of the aneurysmal sack from which the biopsy was derived. In addition, the growth rates of 10/18 AAA patients were not available, partially due to rupture (5/10). As non-ruptured aneurysms are mostly asymptomatic, they are often diagnosed incidentally^3^, which accounts for the other unavailable growth rates due to short follow up time until surgery (5/10). Potential selection bias could have occurred in our study, as we have included all aneurysm patients, who underwent open AAA surgery at the time of the conduction of the study consecutively. However, there were no additional criteria or patient selection, which prevented selection bias. As we cannot regulate the site of aortic biopsies, variation of aortic tissue could cause a bias in the results. The site could not be strictly defined upfront as it was secondary to patient safety. However, all the biopsies came from the abdominal region of the aorta and from a part which belonged to the aneurysmal sac, thus from a diseased, dilated region. Most biopsies were taken from the ventral site of the aneurysm.

We initially hypothesized that SMC contraction *in vitro* might be linked to the degeneration or decrease of SMC specific markers as result of a phenotypic switch^26^. SMC phenotypes have been functionally classified into “synthetic” and “contractile”, characterized by high expression levels of SMC specific contractile proteins; especially smoothelin^27^. The SMC phenotypic switch might also play an important role in AAA formation, due to the loss of the SMC contractile properties, as indicated by findings in a rat animal model of thoracic aortic aneurysms^28^. However, no differences in SMC marker expression or proliferation have been found on mRNA level between patients and controls in our study. Additionally, our results show no correlation between SMC contractility and SMC marker expression. In accordance to our findings, it has been reported that lower contractility of Marfan mice thoracic aortas was not accompanied by a reduction in alpha smooth muscle actin expression^23^.Remarkably, the variability in SMC marker expression is equally present in the control as well as in the patient group. This indicates that SMC contractility *in vitro* is not solely defined by the contractile state of the SMC phenotype.

Endoleaks are defined as a post-operative complication following endovascular graft placement, which is characterized by persistent blood flow into the aneurysmal sac around the graft^29^. Endoleaks can be associated with progressive aneurysm growth and risk of secondary aneurysm rupture^30^. As hinted by previous research^31^, the secondary aneurysm might grow around the positioned graft due to endotension and pressurization by the thrombus surrounding the graft. Based on our findings (Fig. 6b), we speculate that secondary aneurysm growth might be associated with SMC contractile dysfunction. Conversely, progressive aneurysm growth and medial degradation could lead to SMC atrophy, which further weakens the wall in a negative feedback loop and the endograft placed against the aortic wall could cause an inflammation process locally, which affect the SMC as well. It is, however, unclear to which extent these processes are related, and larger scale studies are required to elucidate their connection in the context of AAA pathophysiology.

Tobacco smoking is one of the most important risks for developing AAA, illustrated by four times higher prevalence of AAA in life long non-smokers compared to smokers^3^. Furthermore, it has been shown that smoking increases aneurysm growth rate by 15–20%^32^. However, no causal link between AAA pathogenesis and smoking has been shown in terms of molecular mechanisms^33^. We demonstrate impaired contractility in a group of current smokers compared to the contractility of control SMC. The patients grouped into current non-smokers consist of patients who never smoked and patients who have stopped smoking prior to their inclusion in our study. The link between current and past smoking was examined in detail in a study by Vardulaki *et al*.^34^. Based on their findings, levels of exposure to tobacco smoking caused a higher risk of AAA than duration of exposure. They demonstrated a decrease in risk of AAA formation in people who stopped smoking compared to current smokers. As indicated by our findings, current tobacco smoking is associated with impaired contraction compared to control SMC. Taking into account that the contractility of the current smoking group has a lower median compared to the current non-smoker group, we speculate that current smoking might have a negative effect on aortic SMC function. Furthermore, and in accordance to the previous study^34^, we reason that the pathological effect of smoking on SMC dysfunction might be reversible in AAA patients, indicating potential treatment targets. The association of smoking and potential contractile problems in AAA patients is an interesting finding; however, the latter needs further investigation in a larger cohort and additional molecular experiments to elucidate a potential mechanistic link.

In conclusion, we provide the first proof of impaired SMC contractility in patients with sporadic AAA. We describe a novel application of ECIS, which can be further used as a medium-throughput screening assay for translational studies. Our current results suggest that more than one third of AAA patients had impaired contractility in the heterogeneous population of sporadic AAA patients. This study indicates an association between decreased contractility and current smoking in AAA patients. Taken together, our findings might lead to better understanding of the dilation processes in the aortic wall and new therapeutic targets. Further research is needed to clarify the role of SMC contractility in AAA and identify new therapeutic targets in AAA treatment.

## Methods

### Patient population

Aortic biopsies were obtained during open aneurysm repair in Amsterdam University Medical Centers, location VU University Medical center, Amsterdam and Westfriesgasthuis, Hoorn, the Netherlands. All patients signed an informed consent to participate in the study. Twenty one AAA patient was included, of which thirteen with non-ruptured AAA (NRAAA1-13) and eight with ruptured (RAAA1-8). Tissue of patients RAAA4 and NRAAA1-3 was collected after open repair after earlier endovascular repair and are marked Ɨ (Table S1). Control aortic biopsies used in this study were obtained from non-dilated aorta of post-mortem kidney donors anonymously. All material was collected in accordance to regulations of the WMA Declaration of Helsinki and institutional guidelines of the Medical Ethical Committee of the VU Medical Center. All the experiments and experimental protocols were performed in accordance with institutional guidelines and approved by the Medical Ethical Committee of the VU Medical Center. Clinical information of controls and patients are shown in Table 1. Age and gender of the control and patient group were reported. Additionally, patient characteristics were reported as follows: aneurysm size, known genetic causes of aneurysms, related comorbidities and medication use.

### Aortic biopsy explant protocol and SMC cell culture

Perioperatively harvested human aortic wall was transported from the operating room to the laboratory in ice cold sterile Custodiol® (Dr. Franz Köhler Chemie GmbH, Alsbach-Hähnlein, Germany). All AAA biopsies were taken during open surgical repair of the aorta, usually at the ventral site of the aneurysm. Control aortic tissue was obtained from heart-beating donors during the kidney transplantation procedure. The non-dilated aorta was attached to the renal artery of the kidney used for transplant. The aortic tissue was afterwards transported in custodial, a tissue preservation medium, which made sure that both the kidney and aorta remain viable, and we transported them in a similar way as the AAA patient biopsies.The biopsies were sliced with sterilized surgical instruments in a sterile Petri dish. From the initial biopsy, the intima (in AAA patients often including atherosclerotic plaque and calcification) and the adventitia layer with the attached perivascular adipose tissue and fibers were removed, leaving solely a compact and uniform medial layer. The tissue slice was further cut into pieces of roughly 1 mm^2^, and 15 pieces of tissue were placed on the top quarter of the bottom of a culture flask (25 cm^2^) containing 1.5 ml culture medium. All biopsies were cultured in 231 medium (Thermo Fisher Scientific, Walthem, MA, USA), supplemented with Smooth Muscle Growth Supplement (Thermo Fisher Scientific), and 100 units/ml penicillin and 100 µg/ml streptomycin (Thermo Fischer Scientific), to provide optimal vascular SMC growth. Cells were cultured in a humidified incubator 37 °C, 5% CO_2_. After 10 days of incubation, cell growth could be observed in the form of cell colonies growing around the tissue sections. The culture medium was refreshed two times per week until the cell population became subconfluent. Subsequently, cells were transferred into a larger culture flask of 80 cm^2^. A confluent population of cells was established in approximately 6 weeks form the day the initial aortic biopsy was collected. Primary SMC were used between passage 1–8 in all experiments.

### Measuring SMC contraction

Electric cell-substrate impedance sensor (ECIS; Applied Biophysics, Troy, NY, USA) is a technique used to quantify the attachment and behavior of adherent cells in real time^35^. Cells are seeded on gold-coated electrodes embedded in special culture plates (Fig. 1a). An alternating current is applied to the cells. As a function of the surface covered by attached cells, the system generates an impedance value. The baseline impedance value generated by cell adhesion is derived from changes in voltage between the measuring and counter electrodes^14^. Data on contraction derived from the ECIS are recorded in a well with a monolayer consisting of several tens of thousands of cells. Cell behavior is detected by ten electrodes on the bottom of the well. The contractile output is derived as a mean of the recording of the ten electrodes at the time of the stimulation. In this way, the data are averaged in the whole well.

Sterile 96 well plate arrays with 10 electrodes per well (96w10; Ibidi, Planegg, Germany) were coated with L- cysteine and subsequently gelatin, according to a previously described array preparation protocol^14^. SMC were seeded in duplicate in the array at a density of 30000 cells/well on a surface of 0.3 cm^2^ in a cell suspension of 200 µl in complete medium. Cells were cultured for 48 hours prior to stimulation, allowing cells to attach and establish a monolayer. By the time of the stimulation experiment, the cells formed a subconfluent monolayer, allowing for cell migration and contraction as shown in Fig. 1b. The attachment and spreading of cells on the electrodes generates a baseline resistance value, composed of cell-cell and cell-matrix contact^36^. The impedance was recorded at a frequency of 4000 Hz.

Cells were stimulated with 10 µg/ml ionomycin (Sigma Aldrich, Darmstadt, Germany), a Ca^2+^ ionophore which induces a contractile response in adherent vascular SMC induced by the influx of extracellular Ca^2+^^17^. Cell recovery upon ionomycin stimulation was tested by tracking the impedance after washing out the stimulus and replacing it with ordinary culture medium.

Contractile responses were measured in duplicate in each experiment, and the measurement was repeated two- or three times per cell line in independent experiments. Mean contractile values thus derived from the means of the 4–6 measurements performed for each cell line. Maximum Contractile values represent the highest noted contractile strength for each cell line among all the experiments. As shown in equation (1), the value of a well filled with culture medium without attached cells (290 Ω) was subtracted from all the results in the final calculation. Individual contractile outputs per well were calculated according to the formula bellow, where C indicates contraction (expressed in % of change compared to baseline) and prestimulation (PrS) and poststimulation (PoS) resistance values respectively (expressed in Ω). Contraction of each well thus equals one minus the ratio between the resistance post- and pre-stimulation post empty well value subtraction. The final values thus represent a ratio between the value post- and pre-stimulation and are expressed as percentages of change.

1 $$C=(1-\frac{(PoS[{\rm{\Omega }}]-290[{\rm{\Omega }}])}{(PrS[{\rm{\Omega }}]-290[{\rm{\Omega }}])}\,)\cdot 100$$

Reproducibility and variation was assessed intra- and inter-experimentally. Interexperimental ECIS contraction measurement reproducibility was tested using a difference Bland-Altman plot^37^. Each data point shows the deviation from the mean among two independent measurements of the same cell line. Data points are shown within or outside the 95% confidence interval.

Due to the novelty of our method in the study of AAA pathology, no published references for expected SMC contractility *in vitro* were available. To characterize the contractile output of the patients, the mean response of the control group was used as a reference point for healthy SMC behavior. Deviant contractility was defined as outcomes more than two standard deviations compared to the mean contractile response of the control group.

### RNA isolation and quantitative PCR

To assess differences between the contractile phenotypes of SMC from controls and patients, the mRNA expression level of SMC-specific markers was performed. Using quantitative PCR (qPCR), the expression of early (*ACTA2, TAGLN* and *VIM*) and late (*CNN1* and SMTN) SMC-specific-marker genes was measured. The proliferative capacity of SMC cell lines was assessed by quantification of *MKi67* gene expression. Firstly, total RNA was isolated using the NucleoSpin RNA kit (Macherey Nagel, Duren, Germany) according to manufacturer’s instructions. Next, first strand cDNA synthesis was performed in a 20 μl reverse transcription reaction using the VILO kit (Thermo Fisher Scientific), adjusted to the concentration of total isolated RNA and according to the instructions provided by the manufacturer. qPCR was then performed to quantify the expression of SMC markers *ACTA2, CNN1, SMTN, TAGLN*, VIM and mammalian proliferation marker *MKi67*. *YWHAZ* and *TBP* were used as housekeeping genes to normalize for the amount of total RNA per sample. All data were normalized using a normalization factor derived from the expression of housekeeping genes. RefSeq gene numbers and primer sequences are provided in Supplementary Table 1. Gene expression was determined by the LightCycler 480 Instrument II (Roche Applied Science, Penzberg, Germany), and the reactions were prepared using Light Cycler SYBR Green IMaster (Roche Applied Science), as previously described^38^.

### Immunostaining and confocal microscopy

To examine overall cell morphology and contractile cytoskeletal properties, immunostainings on representative samples of both the control and patient group were performed. Cells were seeded on glass coverslips (#1, Ø 13 mm; Thermo Fisher Scientific) in a seeding density of 100000 cells/ml. The cells were cultured overnight, washed with EBSS and fixed for 10 minutes on room temperature in 4% paraformaldehyde. Next, coverslips were washed 3x with PBS-Tween (PBST) and the cells were subsequently permeabilized for 10 minutes in 0.2% Triton in PBS. Consequently, samples were incubated in blocking solution, consisting of 1% BSA solution in PBST for 1 h at room temperature. Primary antibody against smoothelin (Thermo Fisher Scientific)was incubated overnight on 4 °C, diluted 1:100 in blocking solution. Samples were washed five times using PBST the next day. Secondary antibody (1:100; Thermo Fisher Scientific), DAPI (1:200; Thermo Fisher Scientific), and Phalloidin (1:200; Thermo Fisher Scientific), were incubated for 1 hour on room temperature in the dark. Slides were closed with Mowiol mounting medium (Sigma Aldrich). Images were acquired using the Nikon A1R (Nikon, Tokyo, Japan) microscope and the corresponding software Nis-Elements C Software (Nikon). Representative images were analyzed and adjusted using Fiji^39^.

To obtain representative images of SMC contraction in real time, cells were seeded in 35 mm glass bottom dishes (Thermo Fisher Scientific) coated with gelatin. After overnight incubation, the cells were placed in an incubation chamber at 37 °C, 5% CO_2._ Cells were stimulated with ionomycin and imaged using Nikon A1R (Nikon) in intervals of 10 s for 1 h.

### Western blot

SMC marker proteins in control and patient cell lines were quantified using western blot. Cells were seeded in 6 well plates at a seeding density of 150.000 cells/well and incubated overnight. The next day, they were washed with Earl’s Balanced Salt Solution (EBSS; Thermo Fisher Scientific) and each well was lysed in 100ul of Nu Page Sample buffer and reducing agent (9:1) (Thermo Fisher Scientific. After boiling, 8ul of each sample was loaded onto either NuPAGE 4–12% Bis-Tris or NuPAGE 3–8% TA gels depending on the size of the protein in question. Western blotting was performed as previously published^38^. Primary antibodies were incubated overnight at 4 °C. Primary antibody against tubulin (1:8000) was used as loading control. Primary antibodies against Calponin (1:4000; Abcam), Smooth muscle actin (1:4000; Dako), SM22 (1:4000; Abcam) were incubated overnight at 4 °C. Secondary antibodies IRDye® 800CW Goat anti-Rabbit IgG and IRDye® 680CW Goat anti-Mouse IgG (1:5000; LI-COR Biosciences). The fluorescent signal was visualized using the Odyssey Infrared Imaging System (Odyssey version 4 software; LI-COR Biosciences). Intensity of bands was quantified using Fiji^39^.

### Correlation of contraction and clinical characteristics

To elucidate SMC contractile function, contractility was correlated with gender and age of patients and controls (Table 1). In AAA patients, contractility was correlated with maximum aneurysm size, smoking, diabetes, hypertension, hyperlipidemia and pre-surgery statin use. Additionally, we compared the contractility of SMC obtained during primary surgery and SMC obtained during open conversion after endovascular aneurysm repair (EVAR) because of endoleak causing the aneurysm to grow (i.e. endoleak repair).

### Statistics and graphs

Data were analyzed with SPSS (version 22, IBM Statistics, Armonk, NY, USA). The datasets were not normally distributed and were thus compared using non-parametric tests. Two groups were compared using the Mann-Whitney U test and multiple groups were compared with Kruskal-Wallis test, using the Mann-Whitney U test as post-hoc analysis. Correlations were tested with Spearman’s Rank correlations. Correlation output was corrected using the Bonferroni correction. Data are presented as box plots with median and range. Tests were considered statistically significant at p < 0.05. Representative curves and difference plots, boxplots and scatterplots were constructed using GraphPad Prism7 (GraphPad. La Jolla, CA, USA).

## Supplementary information

Video 1

Supplementary dataset 1

## Data Availability

The datasets generated during and analyzed during the current study are available from the corresponding author on reasonable request.
