# Supplementary material for: Impaired smooth muscle cell contractility as a novel concept of abdominal aortic aneurysm pathophysiology
Source: Sci Rep. 2019 May 2;9:6837. doi: 10.1038/s41598-019-43322-3 (PMC6497672; doi:10.1038/s41598-019-43322-3)

**Supplementary Data**

**Impaired smooth muscle cell contractility as a novel concept of abdominal aortic aneurysm pathophysiology**

Natalija Bogunovic^a,b,c^, MSc, Jorn P. Meekel^a,b^, MD, Dimitra Micha^c^, PhD, Jan D. Blankensteijn^a^, MD, PhD, Peter L. Hordijk^b^, PhD, Kak K. Yeung^a,b,*^, MD, PhD

From the Amsterdam University Medical Centers, location VU University Medical center, Departments of ^a^Vascular Surgery, ^b^Physiology and ^c^Clinical Genetics, Amsterdam, the Netherlands

Running title: Smooth muscle cell contraction in aortic aneurysms

# Supplementary Legends

Supplementary Table S1. qPCR primer information GeneBank sequences and forward and reverse qPCR primer sequences of analyzed housekeeping, SMC marker and SMC regulatory genes.

Supplementary Figure 1. **Representative images of Smoothelin and F-actin immunostaining in control and patient SMC.** Column A: Smoothelin immunostaining depicted in green on the merged image. Column B: F-actin immunostaining depicted in red on the merged image. Column C: merged image of smoothelin, F-actin and DAPI. Scale bar: 50µm.

Supplementary Figure 2. **SMC marker gene expression in control and AAA patient SMC*.*** Panels a-c: Gene expression of *SMTN*, *VIM* and *MKi67* in mRNA isolated from control (▲; n=4), normal contracting (●; n=13) and low contracting SMC (○; n=5). Boxplots are shown as median with range.

Supplementary Figure 3. **Cropped and full-length western blot used in Figure 5.** a) Figure 5a, cropped and grouped lanes from a full western blot. Legend Figure 5a): a)Western blot analysis of aSMA, Calponin and SM22 in Control (n=3), Normal Contracting (n=5) and Low Contracting (n=4) AAA patient SMC. Lanes were cropped and grouped from the original image (Supplementary Figure 3), Ladder: lane 0; Control: lanes 1,4 and 5; Normal contracting: lanes 6-9 and 14; Low contracting: lanes 10-13. b) Intensity of aSMA, Calponin and SM22 in Control (n=3), Normal Contracting (n=5) and Low Contracting (n=4) AAA patient SMC. Intensity red channel (700CW) 5, Intensity green channel (800CW) 3.b) Full-length blot. Intensity red channel (700CW) 5, Intensity green channel (800CW) 3.

Supplementary Video 1. **Time-lapse recording of SMC contraction.** SMC were seeded in a 25mm dish and stimulated with ionomycin. The cells were recorded for 1h and the video was fast-forwarded 900x. Scale bar: 50µm.

Supplementary Table S2. **qPCR primer information GeneBank sequences and forward and reverse qPCR primer sequences of analyzed housekeeping, SMC marker and SMC regulatory genes.**

| Gene code | RefSeq sequence | Forward and reverse primer sequence |
| --- | --- | --- |
|  |  |  |
| *YWHAZ* | [NM_145690](http://www.ncbi.nlm.nih.gov/nuccore/NM_145690) | GATGAAGCCATTGCTGAACTTG |
|  |  | CTATTTGTGGGACAGCATGGA |
|  |  |  |
| *TBP* | [NM_003194](http://www.ncbi.nlm.nih.gov/nuccore/NM_003194) | AGTTCTGGGATTGTACCGCA |
|  |  | TCCTCATGATTACCGCAGCA |
|  |  |  |
| *VIM* | [NM_003380](http://www.ncbi.nlm.nih.gov/nuccore/NM_003380) | AGATGGCCCTTGACATTGAG |
|  |  | CGTGATGCTGAGAAGTTTCG |
|  |  |  |
| *MKI67* | [NM_002417](http://www.ncbi.nlm.nih.gov/nuccore/NM_002417) | AGCACCAGAGGAAATTGTGG |
|  |  | TTTTCAGGGACCGAGTCTTG |
|  |  |  |
| *AIM2* | [NM_004833](http://www.ncbi.nlm.nih.gov/nuccore/NM_004833) | GCTGCACCAAAAGTCTCTCC |
|  |  | ATCTCCTGCTTGCCTTCTTG |
|  |  |  |
| ACTA2 | [NM_001141945](http://www.ncbi.nlm.nih.gov/nuccore/NM_001141945) | ACTGGGACGACATGGAAAAG |
|  |  | CATACATGGCTGGGACATTG |
|  |  |  |
| CNN1 | [NM_001308341](http://www.ncbi.nlm.nih.gov/nuccore/NM_001308341) | GCCCAGAAGTATGACCACCA |
|  |  | TGATGAAGTTGCCGATGTTC |
|  |  |  |
| SMTN | [NM_001207018](http://www.ncbi.nlm.nih.gov/nuccore/NM_001207018) | TGGAGGAATTGACTGCACTG |
|  |  | GAAACCTCTGCCTGCTGTTC |
|  |  |  |
| TAGLN | [NM_001001522](http://www.ncbi.nlm.nih.gov/nuccore/NM_001001522) | AAGAATGATGGGCACTACCG |
|  |  | AGCCCTCTCCGCTCTAACTG |

Supplementary Figure 1.


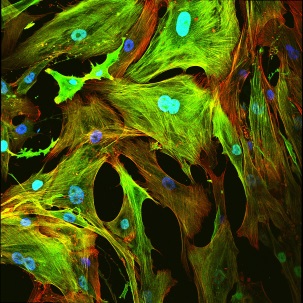

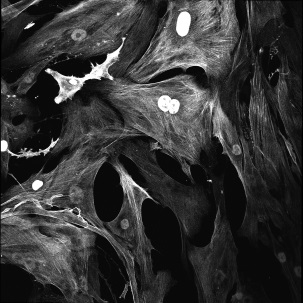

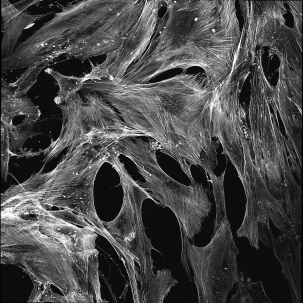

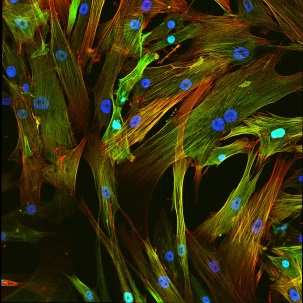

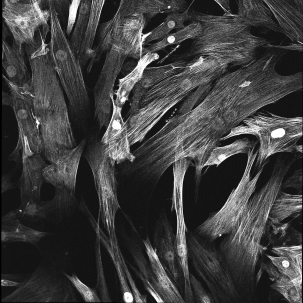

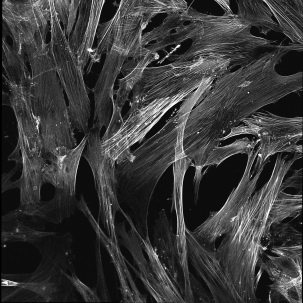

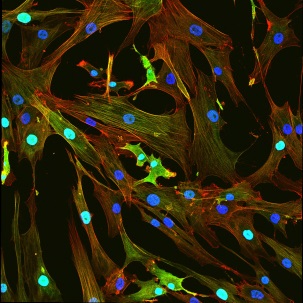

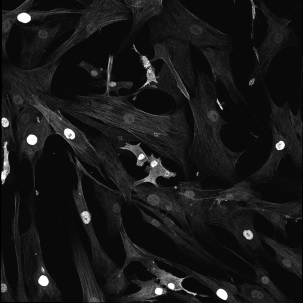

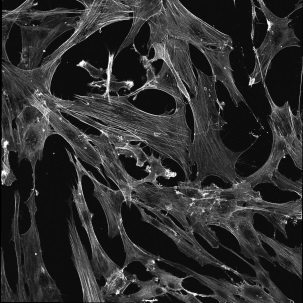

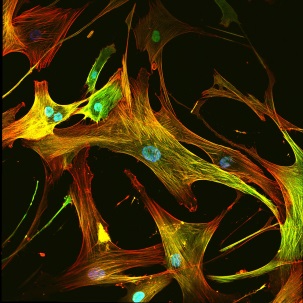

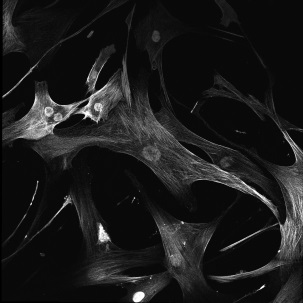

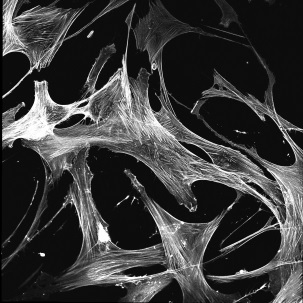

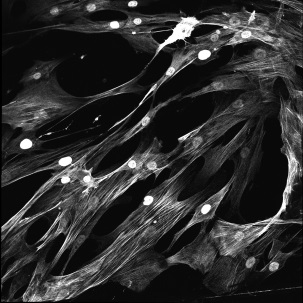

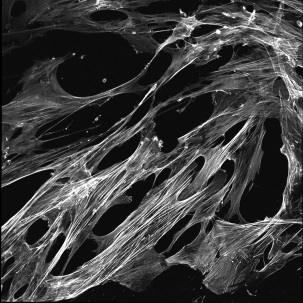

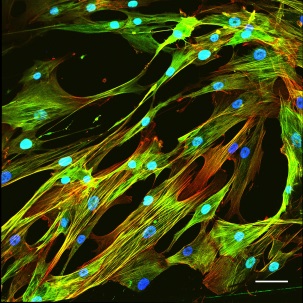


**Control**

Contraction: 70%

**Smoothelin**

**(green)**

**F - actin**

**(red)**

**Merge**

**Control**

Contraction: 69%

**Non-ruptured**

Contraction: 15%

**Ruptured**

Contraction: 20%

**Non-ruptured**

Contraction: 27%

Supplementary Figure 2.

a

b

c

Supplementary Figure 3.


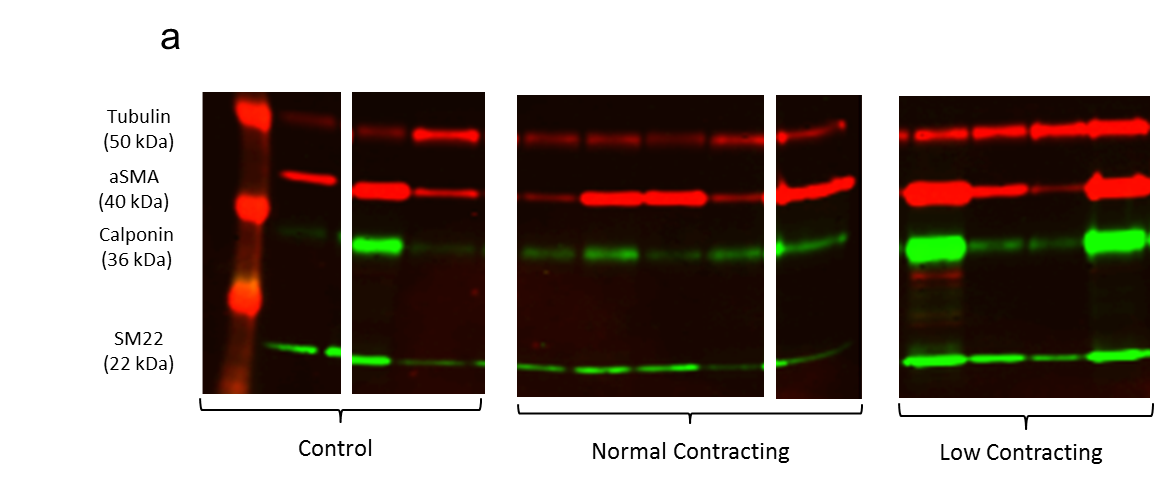


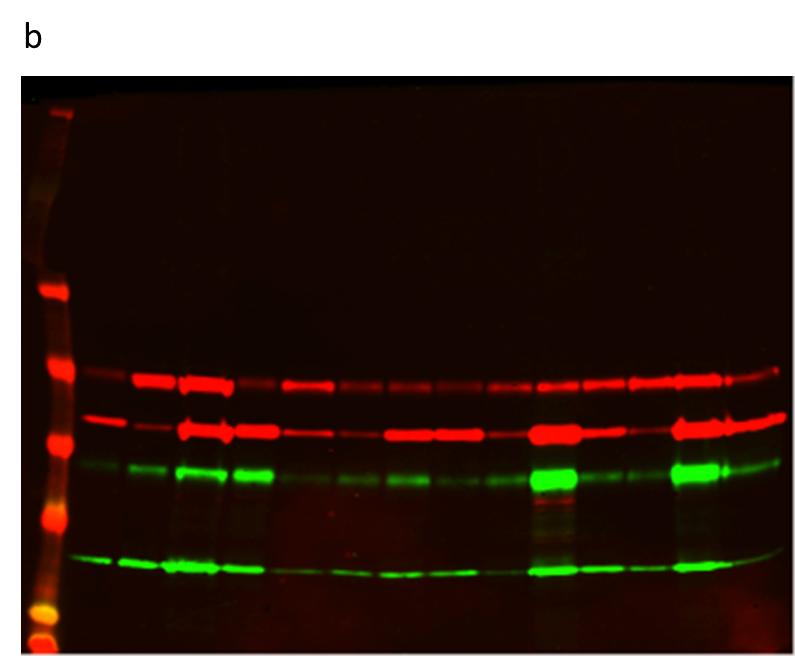

Supplement: Supplementary file 2 — Supplementary dataset 1 [file 41598_2019_43322_MOESM2_ESM.docx]
